# The Global Landscape of EBV-Associated Tumors

**DOI:** 10.3389/fonc.2019.00713

**Published:** 2019-08-06

**Authors:** Claire Shannon-Lowe, Alan Rickinson

**Affiliations:** Institute for Immunology and Immunotherapy, The University of Birmingham, Birmingham, United Kingdom

**Keywords:** Epstein-Barr virus, lymphoma, carcinoma, lymphoproliferative diseases, latency

## Abstract

Epstein-Barr virus (EBV), a gamma-1 herpesvirus, is carried as a life-long asymptomatic infection by the great majority of individuals in all human populations. Yet this seemingly innocent virus is aetiologically linked to two pre-malignant lymphoproliferative diseases (LPDs) and up to nine distinct human tumors; collectively these have a huge global impact, being responsible for some 200,000 new cases of cancer arising worldwide each year. EBV replicates in oral epithelium but persists as a latent infection within the B cell system and several of its diseases are indeed of B cell origin; these include B-LPD of the immunocompromised, Hodgkin Lymphoma (HL), Burkitt Lymphoma (BL), Diffuse Large B cell Lymphoma (DLBCL) and two rarer tumors associated with profound immune impairment, plasmablastic lymphoma (PBL) and primary effusion lymphoma (PEL). Surprisingly, the virus is also linked to tumors arising in other cellular niches which, rather than being essential reservoirs of virus persistence *in vivo*, appear to represent rare cul-de-sacs of latent infection. These non-B cell tumors include LPDs and malignant lymphomas of T or NK cells, nasopharyngeal carcinoma (NPC) and gastric carcinoma of epithelial origin, and leiomyosarcoma, a rare smooth muscle cell tumor of the immunocompromised. Here we describe the main characteristics of these tumors, their distinct epidemiologies, histological features and degrees of EBV association, then consider how their different patterns of EBV latency may reflect the alternative latency programmes through which the virus first colonizes and then persists in immunocompetent host. For each tumor, we discuss current understanding of EBV's role in the oncogenic process, the identity (where known) of host genetic and environmental factors predisposing tumor development, and the recent evidence from cancer genomics identifying somatic changes that either complement or in some cases replace the contribution of the virus. Thereafter we look for possible connections between the pathogenesis of these apparently different malignancies and point to new research areas where insights may be gained.

## Epstein-Barr Virus: Founder Member of the Human TUMOR Virus Group

Epstein-Barr virus (EBV) was discovered in 1964 at a time when oncogenic viruses were widely considered to be a peculiarity of experimental animal models with little if any relevance to human cancer. Its discovery came as a direct result of Denis Burkitt's seminal work (1) identifying a tumor that was the commonest malignancy of childhood in equatorial Africa yet, at the time, was unknown in the West. Such unusual geographic restriction raised the possibility that an infectious agent, perhaps limited to equatorial regions, was somehow involved in the tumor's pathogenesis. Once Tony Epstein and his co-workers Yvonne Barr and Bert Achong had managed to establish cell lines from the tumor, electron microscopy revealed the presence of herpesvirus particles in a small fraction of the cultured cells (2). That virus, EBV, is now recognized as the founding member of the human tumor viruses, and the cancer of African children, now known as “endemic” Burkitt Lymphoma (BL), the first of an ever lengthening list of virus-associated human tumors.

Achieving that recognition was not straightforward, however, and the significance of EBV's association with BL remained in doubt for several years. In particular, sero-epidemiologic studies found that EBV was not restricted to regions of BL endemicity but widespread in all human populations; indeed the virus is carried by most people worldwide as a lifelong asymptomatic infection, behavior that at the time seemed antithetical to tumor virus status. Yet, crucially, molecular studies confirmed that the virus genome was present in all endemic BLs (3), even those from a second area of high tumor incidence in Papua New Guinea (4), strongly suggesting that the EBV/BL linkage was no accident. Reconciling these two contrary aspects of the virus' behavior, ubiquity in its host population yet consistent association with a specific tumor type, remains a fascinating challenge not just for EBV, but more widely for all of the now-known human tumor viruses. Table 1 lists those viruses in chronologic order of their discovery and gives their associated tumor types; they include hepatitis B virus (HBV), human T-lymphotropic virus 1 (HTLV1), the “high risk” human papilloma virus (HPV) subtypes, hepatitis C virus (HCV), Kaposi Sarcoma-associated herpesvirus (KSHV), and Merkel cell polyomavirus (MCV). As discussed fully elsewhere (8), all of these agents establish persistent infections which often seem harmless, yet infection substantially increases the chance of cancer arising in the virus' principal target tissue. Moreover, in all cases except the two hepatitis viruses (which operate indirectly to enhance oncogenic change), the viral genome is present and transcriptionally active in every tumor cell, strongly implying a direct and continuing viral role in tumor growth.

**Table 1:** Human tumor viruses and their associated malignancies.

**Virus (date of discovery)**	**Target cell**	**Associated malignancies**	**Global numbers/yr**
Epstein Barr virus (1964)	B cell	B lymphoproliferative disease	2,000
		Hodgkin lymphoma	28,000
		Burkitt lymphoma	7,000
		Diffuse large B cell lymphoma	15,000
	Epithelial cell	Nasopharyngeal carcinoma	80,000
		Gastric carcinoma	82,700
	T and NK cells	T/NK-lymphoproliferative diseases	<5,000 ^*^
		T/NK lymphomas/leukaemias	<5,000 ^*^
	Smooth muscle	Leiomyosarcoma	<100 ^*^
Hepatitis B virus (1967)	Hepatocyte	Hepatocellular carcinoma	410,000
Human T-cell lymphotropic virus type 1 (1980)	T cell	Adult T-cell leukemia and lymphoma	3,000
Human papillomavirus, high risk types (1983)	Keratinocyte	Genital cancers	598,500
		Head and neck cancer	37,200
Hepatitis C virus (1989)	Hepatocyte	Hepatocellular carcinoma	170,000
Kaposi's sarcoma associated herpesvirus (1994)	B cell	Primary effusion lymphoma	6,500
	Endothelial cell	Kaposi's sarcoma	44,200
Merkel cell polyomavirus (2008)	Merkel cell	Merkel cell carcinoma	<2,000^*^

*Human tumor viruses, listed in their order of discovery, are shown alongside their main target cell type(s), their associated malignancies and the estimated global incidence (new cases arising worldwide per year) of those malignancies*.

* *Incidence figures are based on values given by the Global Cancer Observatory (gco.iarc.fr/causes/infections/home), Parkin (5) and de Martel (6) and, for EBV, updated by Cohen et al. (7). Figures for the rarer malignancies are uncertain, and are shown here as below an estimated maximum*.

Clearly the emergence of a virus-associated cancer offers no evolutionary advantage to either virus or host, but instead represents a subversion of the usual virus-host relationship. Virus infection represents one step in a complex chain of chance events that occur within the pre-malignant cell pool and eventually lead to the emergence of a malignant clone. Despite the complexity of their pathogenesis, virus-associated cancers are not rarities. Collectively they are estimated to account for around 1.3 × 10^7^ new cases of cancer arising globally each year, representing at least 10% of the world's total annual cancer incidence (5, 6). In the long term, vaccination and/or anti-viral drugs have the potential to reduce those numbers but, until then, virus-associated cancers will continue to impose a huge burden on world health.

Epidemiological data (5, 6) suggest that currently some 500,000 of the above 1.3 ×10^7^ cases are attributable to the high risk HPVs, and a similar total to the combination of HBV plus HCV. Next comes EBV with, it is now estimated, around 200,000 cases per year (7), while KSHV, HTLV1, and MCV make smaller contributions to the overall total. Table 1 shows the estimated numbers and the identity of the individual virus-associated tumors. Note that EBV's impact is not only substantial in numerical terms but also unique in the unexpectedly wide range of tumor types with which the virus is linked. All of the other tumor viruses are associated with malignancies arising in cells of their main target tissue. Thus, the high risk HPVs naturally infect genital or sometimes oropharyngeal epithelium and produce carcinomas at those sites; the hepatitis viruses are almost exclusively linked to hepatocellular carcinoma (a proposed HCV/lymphoma link being a possible exception), MCV is a common skin commensal and is found in the Merkel cell skin carcinoma, and HTLV1's T cell tropism is reflected in the origin of HTLV1-associated adult T cell leukemia. Even in the case of KSHV, a gamma 2-herpesvirus linked to three different tumor types, those tumors arise from vascular endothelium (Kaposi Sarcoma, KS) and from B cells (primary effusion lymphoma, PEL, and Multicentric Castleman's disease), cell lineages that are the natural targets of lytic and latent KSHV infection, respectively (8).

By contrast, EBV is causally associated with two lymphoproliferative diseases (B-LPD and T/NK-LPD) and at least eight pathogenetically distinct tumors; these are BL, Hodgkin Lymphoma (HL), Diffuse Large B cell Lymphoma (DLBCL), plasmablastic lymphoma, (PBL), T/NK cell lymphomas, nasopharyngeal carcinoma (NPC), gastric carcinoma, and leiomyosarcoma, plus a ninth (PEL) in which, where present, the virus is always co-resident with KSHV (9). Five of these, including PEL, originate within the B cell system which is the natural reservoir of EBV latency. However, the others are derived from the T and/or natural killer (NK) cell lineage, from nasopharyngeal epithelium, from gastric epithelium and from smooth muscle. The extent to which these non-B cell tissues acquire the virus during EBV's natural persistence *in vivo* remains unclear. There is increasing evidence that some limited entry into the T and NK cell lineages is more common than once thought (10–12); however the lack of virus replication in these cells suggests that they represent cul-de-sacs of latent infection rather than an alternative reservoir of transmissible virus. As to the other non-B cell types above, nasopharyngeal epithelium is the most likely candidate for natural infection since the virus clearly can replicate in oral/lingual epithelium. However, there is still surprisingly little evidence for nasopharyngeal foci of infection being a normal accompaniment of the EBV carrier state. Even in the case of oral/lingual epithelium, the EBV infection appears to be entirely lytic (13–15) rather than establishing a reservoir of latency; indeed it is significant that, despite naturally infecting oral epithelium, EBV is never found in oropharyngeal carcinomas. It is therefore possible that EBV's presence in the nasopharyngeal tumor may stem from a rare acquisition of the virus by cells at this site (see later) rather than reflecting frequent infection of normal nasopharyngeal tissue; the same argument can be made even more forcefully for the EBV-positive tumors arising from gastric epithelium and smooth muscle cells.

The present chapter, giving an overview of EBV biology in the natural host and a brief introduction to the various EBV-associated tumors, is given as background to the more detailed chapters that follow dealing with the individual tumor types.

## The Biology of EBV Infection: an Overview

**Figure 1:**
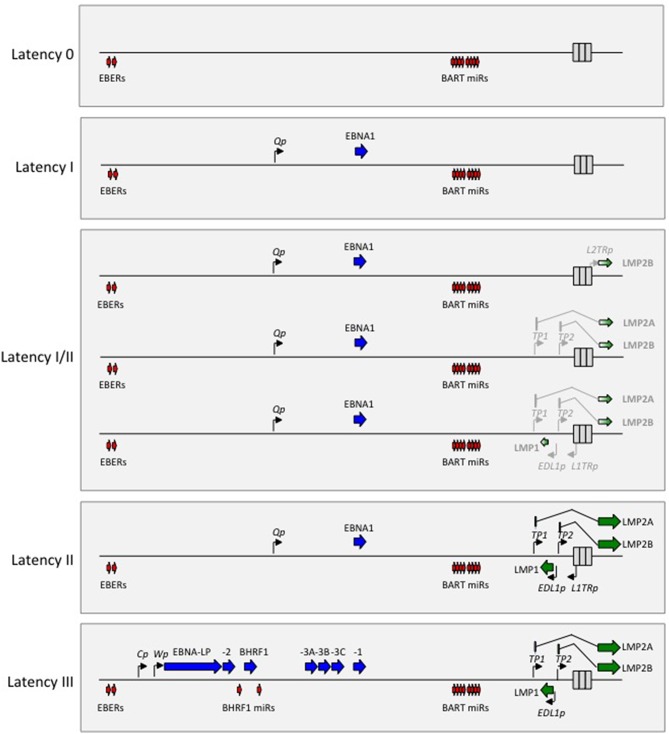
Diagrammatic representation of the alternate forms of EBV latency drawn on a linear map of the virus genome. Note that the non-coding EBER RNAs and the BART miRs are expressed in all forms of latency. Latency 0 represents an antigen-negative form of infection. Latency I involves selective expression of EBNA1 from the Bam H1Q promoter (Qp). Latency II involves expression of EBNA1 from Qp, LMP1 from the LMP1 promoter (EDL1p), LMP2A from the LMP2A promoter (Tp1) and LMP2B from the LMP2B promoter (Tp2). Latency I/II includes a number of latency profiles that are intermediate between Latencies I and II with different degrees of LMP1 and/or LMP2A and/or LMP2B expression (represented here by light shading); in some cases LMP2B is expressed from a different promoter (L2TRp) in the terminal repeat region. Latency III involves expression of all six EBNA proteins, BHRF1 and the BHRF1 miRs from the BamH1W (Wp) and/or BamH1C (Cp) promoters, plus LMP1, 2A, and 2B expressed from EDL1p, Tp1, and Tp2, respectively.

EBV is the human member of the gamma-1 herpesvirus genus, a set of closely related agents that retain the pan-herpesvirus characteristic of life-long latency in the host. However, these agents are distinct from all other herpesviruses in two important respects. Firstly, viewed within the long timescale of herpesvirus evolution, the gamma-1 viruses are the most recent genus and are found only within primate hosts (16); secondly, during that evolutionary process they have acquired a unique set of latent “growth-transforming” genes whose coordinate expression allows the virus to drive its major target cell, the resting B lymphocyte, into proliferation (17). This growth-transforming function is fully recapitulated *in vitro*, as exemplified by EBV which drives normal resting human B cells into permanent virus genome-positive lymphoblastoid cell lines (LCLs). Such LCL cells display a Latency III form of infection, with a transcription programme illustrated on a linear map of the genome in Figure 1. This involves expression of the virus-coded nuclear antigens EBNA 1 (the virus genome maintenance protein), EBNAs 2, 3A, 3B, 3C, and –LP (transcriptional activators/regulators), the latent membrane proteins LMPs 1 and 2A/2B (signal transducers) and low levels of BHRF1 (a bcl2 homolog), plus the non-coding EBER RNAs (EBERs) and two blocks of microRNAs (the so-called BHRF1- and BART-miRs).

Growth-transforming Latency III infection has long been a subject of intense study but it reflects only one of several alternative forms of latency. These alternative forms are shown in Figure 1 in an order that reflects their increasingly restricted transcriptional programmes compared to Latency III. Of these, Latency II (expressing EBNA1, LMP1, LMP2A/2B plus the EBERs and BART-miRs) and Latency I (expressing EBNA1 plus the EBERs and BART-miRNAs) represent forms of infection first identified in EBV-positive tumors, HL, and BL, respectively (9). However, they almost certainly reflect transcriptional programmes that are adopted by the virus at key points during its lifelong persistence within the B cell system (see below). Likewise the concept of a Latency 0 infection (expressing only the EBERs and BART-miRs) is directly inferred from the fact that non-proliferating, EBER/BART-miR-positive but virus antigen-negative B cells constitute the major reservoir of latent infection in the blood of healthy virus carriers (18). With respect to Figure 1, it has become convention in the field to classify EBV-associated malignancies tumors using the Latency I, II, III paradigm; but unfortunately this is an over-simplification. There is a clear distinction between Latency III on the one hand and Latencies I and II on the other based their use of different EBNA promoters (Cp/Wp vs. Qp, Figure 1). However, the Latency I and II profiles themselves, represented respectively, by BL (100% tumor cells EBNA1+) and HL (100% tumor cells EBNA1+, LMP1+, and LMP2+), should be viewed as the extreme ends of a spectrum that contains many intermediate forms. Thus, in some of the other EBV-positive tumors, EBNA1 plus only one of the LMPs (typically LMP2) is expressed, while in others both LMPs are expressed but only in a fraction of the tumor cells. Such tumors tend to be mistakenly categorized as Latency II even though they are far away from the defining HL profile. To correct this, later in this article as in earlier reviews (19), we use the term Latency I/II to designate these intermediate forms.

As described above, EBV:B cell interactions have been exhaustively studied in tumors and *in vitro* models. However, our understanding of the interactions underpinning virus infection in the healthy human host remains limited. The need for informative animal models of *in vivo* infection has therefore long been apparent. Given the similarities between all members of the gamma-1 genus, experimental infection of virus-naïve rhesus monkeys with the rhesus gamma-1 herpesvirus is a model with huge potential (20) but its exploitation is limited by ethical as well as logistic issues. The impasse has prompted efforts to develop a small animal model involving EBV infection of “humanized mice,” i.e., immunocompromised mouse strains whose immune system has been reconstituted by adoptive transfer of human haemopoietic stem cells (21). This has proved useful in studying the acute B-lymphoproliferative disease induced by EBV challenge, and some aspects of the acute T and NK cell responses to that challenge. However, a lack of memory B cell maturation in these animals currently limits their usefulness as authentic models of EBV latency/persistence. At present therefore, our understanding of virus biology is still largely derived from studies of natural EBV infection in the human host. In that regard, most information on primary infection comes from individuals who first acquire the virus in adolescence and develop infectious mononucleosis (IM), rather than from the more usual circumstance of asymptomatic virus acquisition in childhood. The relationship between these two situations remains unclear (22). Likewise, many studies of persistent infection draw heavily on individuals in whom immune impairment has tipped the virus-host balance in favor of the virus, and such imbalance may distort the true picture. These caveats need to be borne in mind when discussing EBV's biology in the natural host.

**Figure 2:**
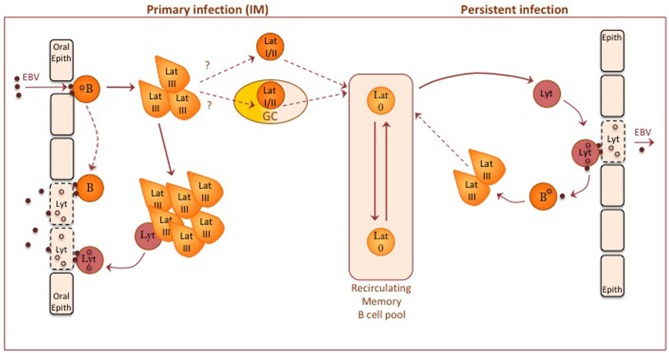
A diagram of EBV-B cell and EBV-epithelial cell interactions thought to occur during primary and persistent EBV infection in the immunocompetent host. Orally acquired virus particles infect both resting B cells and oral epithelium. Current evidence suggests that oropharyngeal B cells may be the initial target, leading to local foci of EBV-transformed B cell growth (via a Latency III- type infection) in oropharyngeal lymphoid tissues; mucosal epithelial cells may then acquire infectious virus either by direct transfer from virus bound to the resting B cell surface or from a small fraction of transformed B cells switching into lytic cycle. Some other virus-transformed B cells are able to down-regulate viral antigen expression, stop proliferating and enter the recirculating memory B cell pool. How this occurs is unclear. One possible route is via a physiologic germinal center (GC) reaction, another via a non-GC route; in either case, this transition may involve progressively more restricted forms of Latency (II and/or I) before entry into Latency 0. Occasional reactivation of latently-infected memory B cells into lytic cycle, possibly induced by triggers of plasma cell differentiation, produces virions that through close cell-cell contact can initiate new latent B cell infections or establish new foci of virus replication in epithelial cells. Although not shown on the diagram, the above events are subject to immune controls; for a description of immune responses to latent and lytic infections, see Taylor et al. (23).

Figure 2 summarizes the basic framework of EBV infection as currently understood, with further detail given in relevant reviews (17, 19, 24). The virus is orally transmitted and is thought to infect two target cell types during primary infection, oral mucosal epithelium and oropharyngeal B cells. With respect to epithelial infection, lytic EBV replication has been seen in differentiating squamous epithelial cells shed into throat washings from IM patients and in oral hairy leukoplakia (13), an oral epithelial lesion found in AIDS patients and other immunocompromised settings. Importantly, leukoplakia biopsies show that this infection is entirely lytic with no evidence of a latent epithelial reservoir (14). While some studies of tonsillar tissues have raised the possibility of latently-infected epithelial cells (25), the prevailing view that *in vivo* latency requires the B cell reservoir is fully supported by the finding that B cell-deficient patients do not establish persistent EBV infection (26).

It is still not clear whether orally transmitted virus first infects epithelial cells or B cells in the naïve host. From *in vitro* models, epithelial cell infection involves integrin binding through the gH/gL dimer of virus envelope proteins, whereas B cell infection is initiated via gp350 binding to the complement receptor CR2 (CD21) and another glycoprotein gp42 (co-opted into the gH/gL complex) binding to HLA II antigen co-receptor (27). Studies of the envelope composition of orally-shed virus, in particular the high content of the HLA II-interacting ligand gp42, strongly suggest that transmitted virus is predominantly epithelial cell-derived and that it will preferentially target B cells (27). In line with this, prospective studies on primary infections leading to IM have found that virus replication and shedding in the throat is not detectable during the long incubation period but appears around the time of onset of symptoms and coincident with the detection of latently-infected B cells in the blood (28). Such findings favor the view that the virus first infects oropharyngeal B cells before seeding foci of lytic replication in permissive epithelium; such spread could occur from *de novo*-infected B cells by direct transfer of surface-bound virions (29) or from latently-infected B cells within early growth-transformed foci then switching into lytic cycle.

The process through which the virus colonizes the general B cell system remains a fascinating but unresolved question (17, 19, 24). As described above, the initial event is believed to be a growth-transforming Latency III infection of oropharyngeal B cells with some limited lytic cycle entry, much as occurs following B cell infection both *in vitro* and in the humanized mouse model. This is followed by the appearance in the blood of latently-infected B cells in which the growth-transforming programme has been extinguished and viral gene expression is limited to the non-coding RNAs (see above, Latency 0); in IM patients these infected cells are initially high in number but gradually subside to low base-line levels typical of the long-term carrier state. A seminally important finding was that, even though both memory and naïve cells appear to be equally infectable *in vitro*, these circulating Latency 0 cells lie exclusively within the memory B cell pool (30). This has led to various possible explanations of EBV's selective colonization of memory. It may be that, while both naïve and memory B cells become infected *in vivo*, only memory cells are able to make the transition to Latency 0; if naïve B cell transformants are locked into a Latency III infection, then they will be removed by T cell surveillance. However, there is as yet no evidence for that scenario. Perhaps more likely is the possibility that EBV-infected naïve B cells are capable of making the switch but, in doing so, are driven to acquire the genotypic and phenotypic features of memory cells. There are at least two potential means of achieving such a naïve to memory switch, one through virus-induced entry into the physiologic route of B cell differentiation into memory, the other by the virus infection actively mimicking the physiologic route. These two possible routes are outlined below and illustrated as part of Figure 2.

**Figure 3:**
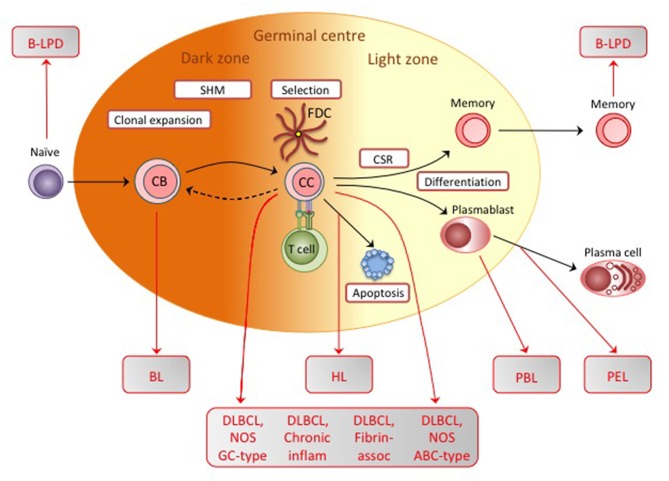
Diagrammatic representation of the germinal center (GC) reaction, showing the physiologic events involved in affinity maturation of antibody responses. Initially antigen-stimulated naïve B cells are induced into clonal expansion as centroblasts (CB), undergoing somatic hypermutation of immunoglobulin genes and producing a centrocyte (CC) population encompassing a range of variant sequences. This is an iterative process and may involve cells undergoing several rounds of CC/CB transition. Most centrocytes then die by apoptosis. Only those expressing immunoglobulin with increased affinity for the original antigen are able to escape by capturing antigen from the surface of follicular dendritic cells (FDCs) and attracting antigen-specific T cell help. Following class switch recombination (CSR) to generate different immunoglobulin isotypes, these centrocytes differentiate either into memory B cells or plasmablasts/plasma cells and leave the GC. Note that, although not shown on the diagram, memory cells in the recirculating pool may be recruited back into a germinal center reaction or directly driven to become plasma cells following re-exposure to antigen. EBV-associated B-LPD and B cell lymphomas display genotypic and/or phenotypic markers that indicate their origin from precursors at one or other position on this B cell differentiation pathway; here we show the presumed identity of those precursors for B-LPD, BL, HL, the various DLBCLs, PBL, and PEL.

The physiologic route into memory involves passage through a germinal center (GC), a complex structure within which affinity maturation of the antibody response occurs (31) (see later, Figure 3). Initially, when antigen-specific naïve B cells encounter and process cognate antigen, they are licensed to receive signals from antigen-specific CD4+ T cells; signals of a particular intensity induce the cells to transform into rapidly proliferating centroblasts in the so-called dark zone of the GC, at the same time undergoing somatic hypermutation of immunoglobulin variable gene sequences (and subsequently class-switch recombination from IgM to IgG or other isotypes). Such proliferation generates, in the light zone, a population of centrocytes, now containing many variant Ig specificities, among which the few cells expressing mutated Ig with improved antigen affinity are selected into memory. Selection occurs via those cells' greater ability to capture antigen from the surface of follicular dendritic cells and, having processed that antigen, then to receive survival signals from antigen-specific CD4+ helper T cells (32); centrocytes failing to compete for antigen lack T cell help and die by apoptosis. It is postulated that an initial Latency III infection of naïve B cells simulates antigen stimulation and induces those cells into a GC reaction (24). Thereafter, in order to survive during the centroblast and centrocyte stages, the cells switch to a Latency II-type with EBNA1 and transient expression of LMP1 and LMP2A. Importantly, LMP1 and LMP2A are, respectively, constitutive activators of the CD40 signaling pathway involved in cognate B/T interactions and of the Ig signaling pathway activated by antigen binding; together their expression could therefore mimic the physiologic survival signals normally required for B cell selection within the GC. As a result, EBV-positive cells would emerge as somatically-mutated, isotype-switched memory B cells which have by that stage extinguished EBV antigen expression (Latency 0), moved to a resting state and joined the recirculating memory B cell pool (24).

The above hypothesis is appealing but there is room for caution here (33). In particular, several groups have looked in tonsils from IM patients, at the very time when the virus is colonizing the B cell system. Using both EBER-RNA *in situ* and latent antigen staining, they find large expansions of EBV-positive cells in tonsillar tissue but these cells are almost all outside GCs in the inter-follicular region (10, 34, 35). Single cell microdissection combined with Ig sequencing shows that such expansions contain several different clonal populations of cells with already-established memory genotypes; interestingly, within each clone there is heterogeneity in terms of latent antigen profiles, with various combinations of restricted latencies present alongside Latency III cells (10, 35). There are a few isolated EBER-positive cells apparently lying within GC structures but microdissection shows that these are but casual immigrants from elsewhere and not part of the adjacent GC population (36).

It is instructive to compare this picture from IM tonsils with that seen in mice following infection with the murine herpesvirus MHV68; this is a gamma-2 herpesvirus which also targets the memory B cell pool but lacks B cell transforming activity and is heavily dependent on GC transit and CD4+ T cell help to establish persistence/latency (37). Unlike the situation in IM, lymphoid tissues from mice undergoing primary MHV68 infection contain many foci of latently-infected B cells actively proliferating within GCs (38, 39). The contrast between the two viruses is again reflected when one compares their infections in hosts that are GC-deficient because of mutation of the X-chromosomal gene, SH2D1A. This gene encodes the SAP protein, a mediator of B cell/T cell interaction that is essential for GC formation (40). MHV68 colonizes the B cell system of SAP-deficient mice very poorly if at all (39); by contrast SAP-deficient humans sustain high levels of EBV infection and may even develop X-linked lymphoproliferative disease in consequence (41). Note that SAP-deficient patients lack conventional isotype-switched memory B cells but, in the absence of GCs, do develop a small non-switched population of IgM+ cells that have low level Ig gene mutation and carry the CD27+ memory marker; interestingly, EBV is selectively found in that population and not in the much larger naïve B cell pool (42). This is further evidence that only B cells with a memory genotype/phenotype are able to carry EBV as a Latency 0 infection *in vivo*. It may be, therefore, that EBV infection/transformation itself can mimic aspects of GC transit without requiring the GC environment; witness the ability of the EBNA3C protein to induce expression of the activation induced deaminase (AID) enzyme essential for somatic Ig hypermutation, and the ability of EBNA3B to elicit B cell/T cell interactions that may be important in delivering environmental cues that can modulate virus gene expression (43). Indeed, central to all these scenarios is the need for the virus to switch between latency programmes in accord with changes in B cell differentiation state, a fascinating subject that is now opening to epigenetic analysis (44).

Clearly there is still much to learn as to how EBV latently-infected cells selectively enter the recirculating memory B cell pool, and also why their numbers remain higher in oropharyngeal lymphoid tissues and in the blood than at other lymphoid sites (45). Once in that pool, one assumes that virus-infected memory cells will be subject to the same physiologic controls that govern the fate of all memory B cells (32). In this regard, re-exposure of memory B cells to cognate antigen can induce one of two physiologic responses, either re-entry into a GC reaction or plasma cell differentiation. Both responses could be exploited by the virus. On the one hand, re-entry into a GC would be expected to amplify latently-infected B cell numbers, and exhaustive screening of tonsillar tissue from long-term virus carriers has detected occasional foci of EBER-positive cells within GCs (12, 46, 47). On the other hand, plasma cell differentiation is known to trigger reactivation from latency into lytic cycle (48). Virus-producing plasmablasts could initiate new infections of neighboring B cells, potentially topping up the latent B cell reservoir, or if situated at a sub-epithelial site as has been seen in tonsillar crypts (12) could seed new epithelial foci of virus replication, thereby increasing the chances of virus transmission to a new host (Figure 1). Within any one individual, the number of EBV-positive cells in the recirculating B cell pool (taken to reflect the latent virus load) appears to be relatively stable over time, but this likely reflects a dynamic equilibrium within which the natural loss of infected cells is balanced by renewal. Interestingly the position of that equilibrium can differ greatly between individual carriers, with the latent virus loads in healthy adults in Western populations spread across a 10,000-fold range (34) and with loads extending even further upwards in populations, for example in equatorial Africa, subject to a range of coincident infections (49, 50). From first principles one might anticipate that individuals carrying naturally high latent virus loads will be at higher risk of developing EBV-associated tumors but, for most of the aetiologically complex tumors, there is as yet no clear evidence that this is the case.

## EBV-associated TUMORS: an Overview

The asymptomatic virus carrier state outlined above is the setting within which most EBV-associated pre-malignant and malignant diseases arise. Table 2 identifies these diseases and their sub-types in more detail, in each case showing the strength of their EBV association (as percentage of lesions that are EBV genome-positive), their Latency profiles and, where known, the identity of co-factors involved in their pathogenesis.

**Table 2:** EBV-associated lymphoproliferations and malignancies.

**Disease**	**Subtype**	**Co-factor(s)**	**% EBV+ve**	**Latency**
B-lymphoproliferative disease	Post-transplant B-LPD, early-onset	Profound T cell suppression	100%	Latency III
	AIDS-B-LPD, late-stage	Profound T cell suppression	100%	Latency III
Hodgkin lymphoma	Mixed cellularity subtype	?	80–90%	Latency II
	Nodular sclerosing subtype	?	15–20%	Latency II
	AIDS-HL; Mixed cellularity subtype	Mid-stage HIV infection	>90%	Latency II
	Endemic BL	Holoendemic malaria	100%	Latency I (or Wp rest^a^)
Burkitt lymphoma	Sporadic BL	?	10–85%	Latency I
	AIDS-BL	Early-stage HIV infection	30–40%	Latency I
	DLBCL NOS (ABC > GC subtype)	Immunosenescence?	10%	Latency II or III
Diffuse large B cell lymphoma	DLBCL-CI	Chronic inflammation	100%	Latency II or III
	FA-DLBCL	Fibrin microenvironment	100%	Latency II or III
	AIDS-DLBCL	Mid-stage HIV infection	30–35%	Latency I or II or III
Other rare B cell lesions	Lymphomatoid granulomatosis	Immune deficiency	100%	Latency II or III
	Mucocutaneous ulcer	Immunosenescence	100%	Latency II or III
Plasmablastic lymphoma		HIV; immune deficiency	80%	Latency I or II
Primary effusion lymphoma		KSHV; late-stage HIV infection	80%	Latency I or I/II
	Chronic active EBV	? Chronic inflammation	100%	Latency I/II
	Hydroa vacciniforme-like LPD	UV light	100%	Latency I/II
	Severe mosquito bite allergy	? Mosquito salivary gland secretions	100%	Latency I/II
T/NK-LPD/lymphomas	Systemic EBV+ T cell lymphoma of childhood	? Chronic inflammation	100%	Latency I/II
	Extranodal NK/T cell lymphoma	? Chronic inflammation	100%	Latency I/II
	Primary nodal T/NK cell lymphoma	? Chronic inflammation	100%	Latency I/II
	Aggressive NK leukemia	? Chronic inflammation	95%	Latency I/II
Nasopharyngeal carcinoma	LEL-type	Genetics/carcinogens/?inflammation	100%	Latency I/II or II
Gastric carcinoma	LEL-type	? Chronic Inflammation	90%	Latency I (or I/II)
	Adenocarcinoma	? *H. pylori*/inflammation	5–10%	Latency I (or I/II)
Leiomyosarcoma	Post-transplant	Profound T cell suppression	100%	EBNA2+/LMP1–^b^
	AIDS-associated	Profound T cell suppression	100%	EBNA2+/LMP1–^b^

*EBV-associated pre-neoplastic/neoplastic diseases, and disease subtype, are shown alongside known or likely co-factors, the percentage of tumors globally that are EBV-positive, and the resident form of EBV latency as defined in Figure 1*.

a *Wp-restricted latency is a variant form not shown in Figure 1. Seen in a small fraction of endemic BLs and associated with the presence of an EBNA2 gene-deleted genome, Wp-restricted tumors express EBNAs1, 3A, 3B, and 3C and the viral bcl2 homolog BHRF1 from the BamHIW promoter Wp (51, 52)*.

b *Viral latency in EBV-positive leiomyosarcoma remains to be fully investigated. Studies to date are limited to latent antigen staining and report expression of EBNA2 in the absence of LMP1*.

### Lymphoproliferative Lesions/Lymphomas of B Cell Origin

All EBV-positive lymphoproliferative lesions/lymphomas of B cell origin can be considered as rare accidents of EBV's normal lifestyle in the B cell system. Almost all display somatically-mutated Ig genes and therefore derive from cells that have been through a GC reaction at some point, either prior to or as part of the oncogenic process. However, these diseases are all distinct entities with differences in epidemiology, histologic appearance, degrees of EBV association and pathogenetic routes. Here we describe their essential features with further details given in recent reviews (53, 54).

#### B-Lymphoproliferative Disease (B-LPD) of the Immunocompromised

The most direct proof of EBV's oncogenic potential comes in the form of the EBV-positive B-LPD that arises in bone marrow and solid organ transplant recipients within the first year post-transplant when their T cell-suppression is most intense (often called “early onset post-transplant LPD”) (53). Children born with primary immunodeficiencies affecting T cell function are similarly at risk (41) Such B-LPD lesions are distinct from the other B lymphomas in at least two respects. Firstly, Ig genotyping shows that some are of naïve B cell origin (54, 55), although most (like the other lymphomas) are derived from memory B cells (55). Secondly, individual lesions can be oligoclonal rather than monoclonal and, where multiple lesions develop in the same patient, they can be clonally unrelated (56). Indeed it is this multi-clonal nature which has led to B-LPD being designated a lymphoproliferation rather than a frank lymphoma, though this should not obscure the fact that cell growth is progressive and the disease is uniformly fatal if untreated. The incidence of B-LPD varies between transplant programmes, being highest in those involving the heaviest immunosuppressive therapy and/or where recipients (typically children) are EBV-naive pre-transplant and subsequently acquire the virus with no residual T cell memory (53).

Note that, before highly active anti-retroviral therapy (HAART), an identical B-LPD lesion was also common in late-stage AIDS patients with profound T cell-impairment (57). The condition, now rare in the post-HAART era, is best designated as AIDS-LPD to distinguish it from other categories of AIDS-associated lymphomas. Note that, in the early AIDS literature, many cases of authentic AIDS-LPDs were reported as “immunoblastic lymphoma” or as “CNS lymphoma,” the latter because in AIDS patients the disease often presented as lesions in the central nervous system.

All such B-LPD lesions display Latency III infection, therefore expressing the full spectrum of EBV latent proteins just as in EBV-transformed B lymphoblastoid cell lines (LCLs) *in vitro* (54). This parallel strengthens the case that B-LPDs represent EBV-transformed B cells growing out in the absence of host immune surveillance. In addition, B-LPD lesions (like LCLs *in vitro*) frequently contain a small fraction of lytically-infected cells, and experiments in immunodeficient mice indicate that cells in early lytic cycle assist virus-transformed B cell outgrowth *in vivo* through secreting paracrine growth factors and angiogenic chemokines (58, 59). Arguably, EBV is both a necessary and sufficient cause of B-LPD, the only other requirement being T cell impairment of the host. This contrasts with most other EBV-positive tumors which typically arise in the absence of profound immune impairment, have a more complex pathogenesis and display more restricted forms of latency.

#### Hodgkin Lymphoma (HL)

HL is an unusual tumor in which the malignant population of Reed-Sternberg cells is massively outnumbered by a marked non-malignant infiltrate, with classical HL being divided into four histologic subtypes based on the nature of the infiltrate, namely the nodular sclerosing (ns-cHL), mixed cellularity (mc-cHL) and the rarer lymphocyte rich (lr-cHL) and lymphocyte-depleted (ld-cHL) subtypes (60). Classical HL occurs worldwide but its incidence is highest in the Western world, where the ns subtype is dominant and where overall rates are elevated by an unusual peak of ns-disease in young adults; indeed it was the similarity between this peak and that seen in the West for IM which led to the idea that ns-HL may be caused by delayed exposure to a common virus, possibly EBV. The logic appeared to be sound, even though the result was not the one expected.

The key evidence emerged in 1989 with the detection in particular tumors of monoclonal EBV genomes that could be localized by DNA-DNA *in situ* hybridization to the Reed- Sternberg cells (61, 62). Subsequent surveys using EBER *in situ* hybridization and LMP1-specific antibody staining confirmed the finding that many cases of classical HL worldwide were indeed EBV genome-positive, with a marked subtype-specific bias. However, it was tumors with mc histology, the majority subtype in many developing countries, that showed the strongest association, with 80–90% tumors EBV-positive; by contrast, <20% ns-disease was linked to the virus, bringing the overall EBV-association rate for HL in the West down to 30–35% (63). Among HIV-positive patients in the West, however, HL incidence is not only raised roughly 10-fold above the general population but almost all these AIDS-HLs are EBV-positive and typically of mc subtype. Interestingly these tumors tend to appear in HIV-infected patients retaining an intermediate level of T cell competence rather than in end-stage AIDS (64). To emphasize its independence of profound immune impairment, the incidence of AIDS-HL has not decreased in the post-HAART era, and may indeed show an increase in the months after commencing HAART therapy (65). A requirement for some level of host immune competence in the development of HL supports the emerging paradigm that, through cytokine cross-talk, Reed-Sternberg cells not only induce their own inflammatory cell infiltrate but are also dependent upon it for continued growth.

The origin of the Hodgkin tumor was long debated because the tumor cells have an anomalous phenotype combining features from different lineages. Microdissection and Ig genotyping of individual Reed- Sternberg cells finally identified HL as a monoclonal tumor of post-GC B cells carrying hyper-mutated, in some cases, functionally crippled Ig sequences (66). However, most phenotypic markers of the tumor's B cell origin have been suppressed during its complex evolution. In that regard, Reed-Sternberg cells present with multiple genetic aberrations and typically display constitutive activation of several cell signaling pathways, typically involving NF-B, JAK/STAT, AP-1 and phosphatidylinositol-3 kinase (PI3K)/AKT signaling (67).

The role of EBV in HL pathogenesis is still not fully understood but current views are discussed in recent reviews (67, 68). It is significant that all EBV-positive tumors consistently exhibit Latency II infection with unusually high levels of the LMP1 and LMP2A proteins maintained in every Reed-Sternberg cell. This is widely taken to infer a continuing role for these proteins in tumor growth, with particular importance ascribed to LMP1's ability to activate several of the signaling pathways mentioned above, in particular NF-kB (69). Support for the idea of LMP1 rendering some genomic changes redundant comes from the observation that cellular gene mutations leading to NF-kB activation tend to be concentrated in EBV-negative cases of the tumor. However, the data are still quite preliminary on this point and a comprehensive genomic analysis of EBV-positive vs. EBV-negative HL is still awaited. Besides a continuing influence on the tumor cell phenotype, EBV may also play a role earlier in HL pathogenesis. Thus, the fact that Reed-Sternberg cells appear to be aberrant survivors of GC transit raises the possibility that, during the evolution of EBV-positive HL, expression of the LMPs may have allowed unscheduled survival of an HL progenitor. Again, there is circumstantial evidence for such a view in that tumors with crippling Ig gene mutation are more commonly seen in EBV-positive cases (70), in line with the known ability of LMP2 to mimic Ig signaling and, in a mouse model system, to avert the deletion of surface Ig-negative cells in the GC (71). Efforts continue to develop mouse model systems that can modulate B cell expression of the LMPs *in vivo* and thereby recapitulate the main events of EBV-associated HL pathogenesis (72).

#### Burkitt Lymphoma

Essentially all cases of the high incidence “endemic” form of BL, as seen in equatorial Africa and in Papua New Guinea, are EBV genome-positive, and the cooperative roles of the virus and of a second co-factor, holoendemic malaria, have been widely discussed in the literature (19, 73–75). In addition, BL does occur in children outside of those areas in a lower incidence “sporadic” form. Incidence rates and EBV association rates for the sporadic tumor vary with geography but in ways that are still poorly documented through lack of good epidemiologic data. It is clear that tumor incidence in affluent Western societies is 100-fold lower than endemic levels and only 10–15% tumors are EBV-positive, whereas data from northern Brazil suggest that sporadic BL is more common than in the West and up to 85% tumors are EBV-positive (76). This prompted the idea that there is a low base-line rate of BL developing independently of EBV in all populations worldwide and that any increases above that baseline reflect the additional impact of the EBV-associated tumor. Yet a third form of BL appeared with the onset of the AIDS epidemic; AIDS-BL presents as an unusually common tumor among HIV-infected adults in the West, reaching an incidence that is much higher even than the endemic disease, and around 30–40% of the tumors are EBV-positive (77). It is still not clear to what extent, if at all, the impact of HIV infection has altered the already high incidence of endemic BL in populations in equatorial Africa.

All three forms of BL share essentially the same defining features. They have a characteristic histology with sheets of monomorphic tumor cells resembling germinal centroblasts interspersed with macrophages, giving tumor sections a starry sky appearance. They have hypermutated Ig gene sequences typical of their GC origin and, most importantly, all carry chromosomal translocations that bring the c-myc gene under the control of either the Ig heavy chain or one of the two Ig light chain loci (76). The resultant deregulation of c-myc gene expression is a major determinant of the highly proliferative BL phenotype. In addition, however, recent genomic analysis largely focussed on sporadic BLs has revealed frequent mutations in the TCF3, ID3, and CCND3 genes affecting cell cycle progression and pro-survival pathways (78). Interestingly, a further extension of that work shows that, compared to EBV-negative BLs, EBV-positive tumors (whether endemic or sporadic) have fewer driver mutations affecting cell survival pathways (79). The implication, that EBV's contribution to the Burkitt cell phenotype is largely anti-apoptotic, accords well with the results from *in vitro* studies. EBV-positive BLs, whether endemic, sporadic or AIDS-related, typically display a Latency I programme and increasing evidence shows that one or more features of Latency I infection (in particular the miRs) promote cell survival, thereby counteracting the apoptotic signals that accompany elevated c-myc expression (80, 81). An anti-apoptotic role for the virus is further emphasized by the discovery of a small subset of endemic BLs with dramatically increased resistance to apoptotic signals; these tumors carry a mutant EBV genome where an EBNA2 gene deletion leads to an aberrant form of virus latency (so called Wp-restricted Latency) characterized by unscheduled, high level, expression of the viral bcl2 homolog BHRF1 (82).

#### Diffuse Large B Cell Lymphomas

Diffuse large B cell lymphoma (DLBCL) is the most common type of high grade non-Hodgkin lymphoma in adults, accounting globally for almost half of all tumors in the non-Hodgkin category. However, DLBCL is itself a heterogeneous group, with two major tumor subtypes. These are defined by gene expression profiling as germinal center B cell-like (GCB) and activated B cell-like (ABC), both of which carry Ig gene mutations reflecting their GC/post-GC B cell origin (83). Remarkably, up to 10% of all DLBCLs are now recognized as being EBV-positive, mostly in lymphomas of the ABC-subtype. The link to EBV was first recognized in 2003 in a study of tumors arising in elderly Japanese patients (84) but it is now clear that EBV-positive disease also occurs, albeit less frequently, in younger people (85). As a result, all EBV-associated DLBCLs are now classified as a separate group, currently called “EBV+DLBCL, not otherwise specified (NOS)” to reflect their distinct pathogenesis (86, 87). Even within this group, however, there is some heterogeneity. Cases arising in elderly patients tend to display Latency III infection, similar to that seen in B-LPD, supporting the view that tumor outgrowth is a consequence of reduced EBV-specific T cell surveillance, part of the general decline in T cell competence that occurs with advancing age (“immunosenescence”). By contrast, EBV-positive DLBCLs in younger patients are more frequently Latency II, implying a more complex pathway of tumor evolution (84–86); for example, if cellular genetic changes in Latency III cells render some aspects of EBV's growth-transforming programme redundant, cells able to switch to more limited EBV antigen expression would have a competitive advantage through evading virus-specific surveillance.

Interestingly, DLBCL is yet another type of B lymphoma whose incidence is significantly increased in HIV-positive individuals. The true nature of these authentic “AIDS-DLBCLs” is becoming clearer now that HAART has greatly reduced the numbers of AIDS-LPD lesions arising in HIV patients. This has revealed a landscape of authentic DLBCLs that can appear at any stage of HIV infection and are EBV-positive in 30–35% cases (therefore still significantly higher than the 10% association rate seen in DLBCLs in the general population). Interestingly these AIDS-DLBCLs can display either Latency I, II or III infection (88). There is a parallel here with the picture that is emerging in studies of long-term solid organ transplant recipients i.e., patients who have gone through their initial period of acute B-LPD risk (associated with strong immunosuppressive therapy) but stay on low level immunosuppression for life. Such patients are at risk of so-called “late onset” post-transplant lymphomas, particularly DLBCLs of which some 30–70% are EBV-positive, again with a range of Latency profiles (54, 89).

As interest in EBV's association with DLBCL had grown, a number of other much rarer DLBCL-like malignancies with links to EBV have been recognized. One of these, now formally termed “DLBCL associated with chronic inflammation (DLBCL-CI)” was first described in patients with a long history of pyothorax (90) following treatment for pulmonary tuberculosis, but has now been observed in other chronic inflammatory situations (68). Such tumors are consistently EBV-positive and display Latency III infection, suggesting that they arise in a chronic setting of locally impaired immune surveillance. A somewhat similar lymphoproliferation, again rare but consistently linked to EBV, has been reported in situations of local chronic fibrin deposition and termed “Fibrin-associated (FA-) DLCBL” (91).

#### Other B Cell Lymphoproliferations/Lymphomas of the Immunocompromised

Finally, there are a number of other rare B cell diseases, either categorized as lymphoproliferations or as frank malignancies, which are EBV-associated and which arise in one or other immuno-compromised setting. Of these, lymphomatoid granulomatosis, a lymphoproliferative lesion arising in the lungs of immunodeficient patients, is characterized by different degrees of EBV-positive B cell expansion against a background rich in small T cells. EBV antigen profiles indicate either Latency III or II infection, in line with histologic grading of the B cell population from LPD-like in low grade lesions to large B cells, many with a Reed-Sternberg-like appearance, in more advanced disease (92). Another more recently identified condition is EBV-positive mucocutaneous ulcer, an indolent lesion of skin or mucosal surfaces first described in elderly patients and now also recognized in other immunocompromised groups. Again this involves EBV-positive B cells, some of which are Reed-Sternberg-like, seen here within a mixed cellular infiltrate (93, 94).

In addition to the above, EBV is also linked to two frankly malignant diseases of plasmablastic origin, both recognized first in late-stage AIDS patients in the pre-HAART era and subsequently in other heavily immunocompromised settings. These are plasmablastic lymphoma (PBL) itself (95), typically presenting in the oral cavity, and primary effusion lymphoma (PEL) (96), a body cavity-based tumor recognized as plasmablast-like despite having lost many other markers of its B cell origin. For both these rare lymphomas, EBV is present in around 80% of cases; all PELs and many PBLs display a typical Latency I infection, but in some PBLs the presence of occasional LMP1-positive cells suggests an intermediate Latency I/II. Most importantly all PELs, irrespective of EBV status, are KSHV-positive and this has raised doubts about EBV's role in disease pathogenesis. However, recent evidence strongly suggests that EBV, where present, is influential. Thus, introducing EBV into a KSHV-positive PEL cell line *in vitro* enhanced KSHV genome maintenance (97), while in the humanized mouse model co-infection with both viruses improved KSHV persistence *in vivo* with dually-infected cells acquiring a plasmablast-like gene expression profile similar to that seen in PELs (98).

### Lymphoproliferative Lesions/Lymphomas of T/NK Cell Origin

The discovery in the early 1990s that EBV was also linked to rare lymphoproliferative lesions/lymphomas of T or NK cells came as a major surprise. Until that time, both *in vitro* and *in vivo* studies of lymphoid cell infection had implied that the virus was strictly B cell-tropic. Today the full spectrum of T/NK cell diseases states is well-documented (68, 99), even if the boundaries between them can sometimes blur. They include three pre-neoplastic lymphoproliferative lesions, the systemic form of chronic active EBV (CAEBV), and two cutaneous forms, hydroa vacciniforme (HV), and severe mosquito bite allergy (SMBA), and at least four distinct malignancies, systemic T cell lymphoma of childhood (STLC), extranodal NK/T cell lymphoma (ENKTL), primary nodal lymphoma, and aggressive NK leukemia (ANKL). Although the number of cases is low in global terms, these diseases affect certain populations preferentially, being commonest in East Asian countries such as Japan and in native South/Central American people (themselves of East Asian origin). Moreover, almost all of these T/NK-derived lesions are highly aggressive, extremely difficult to treat and have a universally poor clinical outcome.

Despite this distinct racial predisposition, as yet no genetic links have been established to account for the increased disease risk and no local co-factors have been identified. Furthermore, these conditions arise in individuals with no evidence of pre-existing immune-defects, implying that T/NK cell proliferation (unlike B-LPD) is not dependent on impaired EBV-specific surveillance but rather that the infected T/NK cells can escape such surveillance. Interestingly, patients with EBV-associated T/NK cell LPDs often first present with the symptoms of haemophagocytic lymphohistiocytosis (HLH) (100), a febrile condition generally associated with cytokine storm which can arise in various circumstances but here reflects acute cytokine release from the infected T/NK cells themselves. Work in an *in vitro* model system suggests that LMP1 expression is the key driver in this context, through NFkB-mediated activation of a pro-inflammatory response that is programmed in T and NK cells (101).

#### Chronic Active EBV, Systemic Form

Studies in the 1980s first drew attention to patients with persistent (>3 months) and severe IM-like symptoms, highly elevated EBV antibody titres and high viral loads in the blood (102). The disease, seen particularly in East Asian populations and typically affecting young children, appeared to be a direct result of primary EBV infection and became known as “chronic active EBV (CAEBV)” (103). It is now classified as systemic CAEBV, to distinguish it from related cutaneous forms (see below). The key discovery of EBV in T cells was first made in a Japanese patient with systemic febrile symptoms that, in retrospect, were consistent with CAEBV (104); this prompted many similar studies including examples of patients with NK cell infection (99) or with coincident infections in both T and NK lineages (105). In recent years systemic CAEBV has been increasingly recognized also in adults, where symptoms are often exacerbated. Although the disease is potentially life-threatening, the clinical outcome can vary significantly between individuals and between adults and children. Some patients can remain stable without therapeutic intervention, whilst others exhibit a rapidly progressive disease with severe complications such as multi-organ failure or development of EBV-positive lymphoma. There are even some CAEBV-like cases in the Western world where the virus is restricted to B cells (106). The reasons for this inter-patient variability remain unclear, although it would be hugely beneficial to stratify patients according to predictors of disease progression. Haematopoietic stem cell transplant is currently the only effective therapeutic option for the condition.

There are increasing efforts to understand EBV's role in the pathogenesis of CAEBV and to identify the additional steps that predispose to malignancy. Progress is now being made in both respects. Infected cells typically display a Latency I/II-like EBV latent antigen profile with LMP1 sometimes present and with LMP2 transcription limited to the LMP2B isoform (107). However, this is not the only peculiarity. Recently, sequencing the EBV genome in infected cells from >70 Japanese CAEBV patients revealed frequent single point mutations (relative to the Japanese Akata strain) and, in around 30% cases, a series of intragenic deletions asymmetrically distributed across the viral genome (108). Interestingly, certain BART miRs that repress lytic cycle entry were often lost (as were several core replication genes) while their BZLF1/BRLF1 immediate early target genes remained intact, as if enhanced activation of an abortive lytic cycle in some cells may favor clonal expansion. In the same study, cell genome sequencing indeed showed that many of the pre-malignant lymphoproliferative lesions were monoclonal. Furthermore, those clones already carried somatic mutations affecting potential driver genes, the most common being the gene encoding the RNA helicase DDX3X which has been implicated in other lymphoma settings (108). This clearly indicates the pre-neoplastic nature of CAEBV with both EBV and cellular gene mutations already in place.

#### Hydroa Vacciniforme-Like Lymphoproliferative Disease/Lymphoma

Hydroa vacciniforme was initially described in 1862 but its identity as an EBV-positive lymphoproliferative lesion was not realized until 1999 (109), a decade after the first reports linking EBV with T/NK cell diseases had appeared. Confusion over disease nomenclature has recently led WHO to re-name the condition HV-LPD. This is a rare ultraviolet light-induced eruption of the skin that occurs during childhood, lacks systemic symptoms and in most cases spontaneously remits after adolescence. However, in some cases the disease progresses, especially in East Asian and native South/Central American patients. Lesions spread to sun-protected areas and this is accompanied by systemic high-grade fever, HLH-like symptoms and, in up to 21% of patients, the development of HV-associated lymphoma (HVL). The tumor cells are usually of CD8+ T cell or NK cell origin and only rarely from CD4+ cells. Though not well-studied, they are apparently classifiable as Latency I/II with LMP1 expression sometimes detectable (110). However, the etiology of the disease remains a mystery. Studies to address the role of EBV, to understand the immunological dysfunction or analyse the genetic background have not yet been performed.

#### Severe Mosquito Bite Allergy

Mainly occurring in Japan, severe mosquito bite allergy is not an allergic disease, rather a rare cutaneous manifestation of CAEBV characterized by hypersensitivity to mosquito bites. Skin lesions begin as erythmatuos papules that develop into bullae and ulcerate, but in many cases eventually heal with scarring. The lesions contain a mixed lymphoid infiltrate of CD4+, CD8+ T cells and NK cells, but within this is a small (<10%) oligoclonal/monoclonal population of EBV-positive cytotoxic NK cells (111). The latency profile is poorly studied but, by analogy with HV-like LPD of NK origin, it is provisionally classified as Latency I/II. Following recovery, patients become asymptomatic until the next mosquito bite. The prognosis is variable; some patients maintain a protracted course of cutaneous manifestations whilst others may progress to systemic CAEBV or develop ANKL.

#### Systemic EBV+ T Cell Lymphoma of Childhood

This extremely rare but highly aggressive disease is characterized by the development of a clonal proliferation of EBV-infected CD4+ or, more commonly CD8+ T cells with an activated cytotoxic phenotype (93, 112). The majority of cases are associated with primary infection of children or young adults, but occasionally may arise later as a progression from severe CAEBV. Patients initially present with IM-like symptoms including fever, general malaise and upper respiratory illness but rapidly develop hepatosplenomegaly, abnormal liver function, pancytopenia and CNS symptoms. The disease is complicated by HLH, coagulopathy, sepsis and multi-organ failure and patients usually die within days to weeks following diagnosis. The EBV latency profile is currently described as Latency I/II, with LMP1 sometimes detectable by staining (93). Cellular genetic changes have not yet been characterized, due to the rarity of the disease.

#### Extranodal NK/T Cell Lymphomas

Extranodal NK/T cell lymphoma (ENKTL) is the most common type of nasal lymphoma in endemic areas, representing up to 10% of all non-Hodgkin lymphomas in particular areas of Asia and Central America, whereas it is much rarer (<1% of all non-Hodgkin lymphomas) in the United States and Europe (113). Most ENKTLs arise in the nasal area, where their highly invasive, tissue-destructive nature led to their original description as lethal midline granulomas. It was through studying the nasal tumor that the link to EBV was first discovered in 1990 (114). However, such tumors can also present at extra-nasal sites (skin, GI tract, testis, lungs etc) with dissemination to multiple organs. Importantly, bone marrow involvement in both forms of disease is uncommon, although HLH is a frequent accompaniment and is often indicative of advanced disease.

Most nasal (85%) and many extra-nasal (50%) ENKTLs are of NK (CD56+, CD3–, CD3ε+ and CD3ζ+) cell origin, with the rest mainly from CD8+ T cells. The tumors display Latency I/II with variable expression of the LMP1 protein (115) which, among its multiple effects, has been implicated in driving the hypermethylated phenotype characteristic of ENKTLs (116). Genomic analysis has indeed found epigenetic silencing of certain cell cycle regulatory genes in the tumor, as well as recurrent mutations and/or deletions most often affecting members of the JAK/STAT signaling pathway (Jak3, STAT3, and STAT5B) and the RNA helicase DDX3X (117, 118).

#### Primary Nodal T/NK Cell Lymphoma

EBV-positive primary nodal T/NK cell lymphoma has been included into the WHO classification as a provisional subgroup of peripheral T cell lymphoma, NOS. Mutational analysis has not yet been performed on these lymphomas but it is already clear that they differ from ENKTL in several respects; thus they are typically of T cell rather than NK cell origin, nasal/extranodal involvement is rare and they lack the nasal tumors' angiodestructive phenotype (119). Neverthleless, these nodal tumors are extremely aggressive with a median survival of 4 months.

#### Aggressive NK Leukemia

ANKL is a very rare and extremely aggressive neoplasm that usually manifests in young adults as fever, pancytopenia, and hepatosplenomegaly, often complicated with HLH. The disease course is fulminant, with multi-organ failure and disseminated intravascular coagulation, death occurring within <2 months. The vast majority of ANKL are associated with a Latency I/II EBV infection of NK cells (120, 121), although rare EBV-negative cases have been described with a clinical course indistinguishable from EBV-positive disease, albeit occurring in older adults.

The tumor cells have multiple karyotypic abnormalities, with frequent chromosomal gains and losses, and common deletion on chromosome 6q21-25. Like ENKTL, recent genetic analysis of ANKL has also revealed a very similar range of mutations, particularly affecting the JAK/STAT signaling pathway and DDX3 (122).

#### Commonality Among the EBV-Associated T/NK Cell Diseases

The T/NK cell diseases have for some years been a neglected area of EBV research, though this is now changing rapidly. It is striking that there is greater commonality among these various diseases that one sees among the LPDs and lymphomas of B cell origin. Virologically, we classify the resident pattern of latency in T/NK lesions as typically Latency I/II to reflect the variable detectability of LMP1 staining and the paucity of data regarding LMP2.However, other non-latent viral proteins may play a role at some stage; in that regard, it will be interesting to see whether the virus genome deletions found in CAEBV lesions (implying an additional contribution from abortive lytic infection) are also seen in the various T/NK lymphomas. With respect to mutations in the cellular genome, the frequency with which similar genes are targeted in both premalignant and malignant T/NK lesions, DDX3 being the best example, implies the existence of shared cellular changes that are required to complement the virus' contribution.

The discovery of EBV's involvement in T/NK disease immediately raised two important but still unresolved questions, namely what is the route of viral entry into these target cells and how often does it happen in a regular EBV infection? In terms of entry, one possible route is through a “virological synapse”; such a pathway, first seen in the HTLV1 system (123), involves virions moving through inter-cellular bridges formed when an immune effector cell, in the present context either a virus-specific T or NK cell, engages its lytically-infected target. However, there is as yet no clear evidence that EBV can extend its tropism in this way. Another interesting possibility stems from the observation that, in some T/NK-LPD patients, EBV can be found in both T cell and NK cell fractions (105, 108), and genomic analysis of such cases now shows that both infected populations have common somatic mutations, indicating a shared origin (108). This suggests that EBV has infected a T/NK cell precursor rather than the mature cell types, giving new emphasis to the long-published finding that CD21, the EBV receptor on B cells, is transiently expressed on human thymocytes before their commitment to the T cell or NK cell lineage (124, 125).

As to the frequency of T/NK cell infection *in vivo*, the assumption for many years was that EBV accessed these cell types only rarely and that such access carried a high disease risk. Recently, however, this view has been challenged by a number of observations. Thus, one group has published the surprising finding that EBV is able to infect T cells as well as B cells, both *in vitro* (126) and in a humanized mouse model (127); furthermore, virus-positive T cells appear detectable in the circulating lymphocytes of young African children following natural infection (11). One possible explanation for the fact that such seemingly obvious T cell tropism had been missed hitherto was the authors' observation that infection was exclusive to EBV strains of the type 2 subset, i.e., strains distinguished from the more common type 1 strains by linked polymorphism of their EBNA2, 3A, 3B, 3C genes. Meanwhile another group has carried out a careful re-examination of tonsillar sections from acute IM patients and observe that, alongside the dominant B cell infection, there are small numbers of EBER-positive, latently-infected T cells present, mainly but not exclusively of the CD8+ subset, and even a few rare EBER-positive NK cells (10). Note, however, that all of these IM patients were European and their resident virus strains were identified as type 1, the type dominant in European and North American populations. The type-specificity of T/NK cell infection therefore remains unclear; indeed recent studies in Japan have confirmed that the EBV strains found in T/NK diseases are predominantly type 1 (108), again the prevalent type in that country. Although some of these more recent findings are contradictory in detail, they have reawakened interest in the possibility of a T/NK cell reservoir of virus infection, and potentially of virus persistence, *in vivo*.

### Tumors of Non-lymphoid Origin

While the present volume is focussed on EBV's association with tumors of lymphoid origin, the main contributors to the global burden of EBV-positive malignancies are actually two epithelial tumors, undifferentiated nasopharyngeal carcinoma and a particular subset of gastric carcinomas. The essential epidemiology of these tumors and their relationship to EBV are described below, along with brief mention of a very rare (and still poorly studied) EBV-positive malignancy derived from smooth muscle cells.

#### Nasopharyngeal Carcinoma (NPC)

The link between EBV and the undifferentiated form of NPC was stumbled upon by chance in 1966 (128) and was the first indication that the virus' oncogenic potential was not confined to the B cell system. The discovery was of especial interest because of this tumor's other unusual features: firstly its distinct histology, with a marked lymphocytic infiltrate giving the tumor a lymphoepithelial-like (LEL) appearance, and secondly its unique epidemiology, with an unusual age peak in 40–60 year old males and strikingly different incidence rates across the world (129). Thus, while rates are low in the West, the tumor occurs at intermediate to high incidence throughout South-East Asia and reaches its peak in populations of Southern Chinese decent. There are also pockets of intermediate to high incidence both in North and East Africa and among the Inuit people of the Arctic; interestingly the latter are also at risk of a salivary gland carcinoma which is very similar to NPC in its appearance and virus association, and is usually considered a variant of the nasal tumor. Irrespective of geography and of incidence rate, all cases of undifferentiated NPC worldwide are EBV-associated, with the viral genome present in every malignant cell. Such is the high frequency of NPC in some of the world's most densely populated areas that this tumor alone is estimated to impose a global burden of around 80,000 new cancer cases per year (5).

At least three factors contribute to NPC risk. Firstly, there is clearly a genetic element in that ~10% of NPC patients in the Southern Chinese population come from families with affected relatives (130); moreover genomic studies on non-familial cases have identified several allelic determinants of susceptibility, most dramatically within the HLA class I region (131, 132). Secondly, studies showing a partial decline of NPC incidence rates in successive generations of Southern Chinese migrant families in the West strongly suggest that risk is also influenced by exposure to a lifestyle/environmental factor in one's early years of life (133, 134); circumstantial evidence for a dietary influence common to Southern Chinese cultures, in particular weaning on salted fish, is strong in that regard (135). Thirdly, there is infection with EBV itself, which in many at-risk populations typically occurs in infancy.

The role played by EBV in the pathogenesis of NPC has long been a subject of investigation (135, 136). Clearly the virus must act in concert with the complex set of genomic changes that are now being identified by whole genome sequencing. Viral gene expression in tumor cells is best described as an intermediate form, Latency I/II. Thus, all tumors express EBNA1, the non-coding EBERs and BART-miRs, and (by transcriptional analysis) LMP2 (135, 136). As to their involvement in pathogenesis, xenotransplantation has suggested an *in vivo* growth-promoting role for the BART-miRs (137), which are present at unusually high levels in NPC (138), while LMP2 is another potential contributor given its ability to promote epithelial cell growth in *in vitro* models (139, 140). The case of LMP1 is particularly interesting because one of its main downstream effects, activation of the NFkB signaling pathway, is a consistent feature of all NPCs (141). However, while LMP1 is consistently expressed in the few EBV-positive pre-neoplastic lesions identified so far in the nasopharynx, the protein is only detectable in around 25% cases of primary tumors (142). This has led researchers to postulate an early pathogenetic role for LMP1, which is retained in some cases but rendered redundant in others. Recent whole genome sequencing has offered circumstantial support for this notion by showing that mutations affecting NFkB pathway genes are a common feature of many tumors (142, 143) but, crucially, are hardly ever seen in those cases in which LMP1 expression is maintained (142).

While EBV infection at oropharyngeal mucosal sites appears to be part of the natural virus-host interaction, the degree to which EBV naturally infects nasopharyngeal epithelium remains in doubt. This raises the question as to when the virus is acquired by the NPC progenitor population during tumor development. Here again cancer genomics has opened a way forward by showing that loss/epigenetic inactivation of the tumor suppressor gene p16, and/or amplification of the gene coding its pro-proliferative target cyclin D are common features of the tumor (144); indeed cytogenetic evidence suggests that similar changes are already present in rare dysplastic lesions of the nasopharynx that have not yet acquired EBV (145, 146). This has prompted insightful studies in a cell culture model showing that, while normal nasopharyngeal cell lines cannot be stably infected with EBV, enforced over-expression of cyclin D allows such cells to retain the viral genome and establish a Latency II infection (147). The implication is that pre-neoplastic changes in nasopharyngeal epithelium, caused by exposure to inhaled particulate carcinogens, establish a pool of target cells that can now sustain latent infection. Such particulate insults could also induce inflammation in the nasopharynx, and indeed there is mounting epidemiological evidence linking a history of chronic rhinitis with greater NPC risk (148, 149). In some circumstances an inflammatory environment can itself promote oncogenic change; in this case, however, by trafficking lymphocytes into the nasopharynx, it could increase the chance of EB virions reactivating from the B cell reservoir and accessing the pre-neoplastic cells. A multi-step map of NPC pathogenesis, based on a synthesis of epidemiologic, genomic and biologic data, has been presented and discussed in recent reviews (135, 150).

#### Gastric Carcinoma

A link between EBV and gastric carcinoma was first intimated in the early 1990s when pathologists began to screen archival tumor material by *in situ* hybridization with EBER RNA-specific probes (151). However, the low numbers of EBER-positive tumors, and the fact that gastric carcinoma already had textbook status as a *Helicobacter pylori*-associated malignancy, meant that these early findings did not get the attention they undoubtedly deserved. Interest finally returned and it is now clear from studies in many countries that up to 10% of all gastric carcinomas are EBV-positive (152, 153). Moreover, just as with NPC, all the malignant cells within an individual tumor are EBV-positive and, by terminal repeat analysis, carry the same monoclonal virus genome. Thus, each tumor has developed from a single EBV-infected progenitor cell and was not casually infected after the malignant state was already established.

Gastric carcinomas as a general group vary in their histology and in their distribution within the stomach, presenting either at proximal (cardia) or, more commonly, distal (non-cardia) sites. At least 75% of all such tumors appear to be linked to a previous or ongoing *H. pylori* infection, the association being even higher for the many tumors arising at distal sites (154). Distinct from this majority, the EBV-associated tumors appear to form a clinically and pathogenetically distinct subgroup. Compared to their EBV-negative counterparts, the virus-positive tumors show a greater male predominance, tend to present earlier in life, more often locate in the cardia/body of the stomach than at distal sites, have a lower rate of lymph node involvement and a relatively favorable prognosis (153, 155). However, they can vary in histological appearance. Interestingly, there is one small histologic subgroup of gastric carcinomas that have a rich lymphocytic LEL-like infiltrate resembling that seen in NPC; indeed this similarity was what prompted the early work looking for a viral association. These LEL-like tumors account for only 1–4% of all gastric carcinomas but are EBV-positive in >90% cases (156). By contrast the vast majority of gastric tumors, often grouped together as “conventional adenocarcinomas” in EBV association screens, are not LEL-like. Remarkably, however, some 5–15% of these more common tumors are also EBV-positive; association rates vary between countries but tend to be slightly higher in Central/South America than in Europe or Asia (157). In absolute terms, therefore, most EBV-positive gastric tumors are not LEL-like but have more conventional histology, though many are reported to show some lymphocytic infiltration (so-called “Crohn's Disease-like reaction”) at the leading tumor edge (158). Whether *H.pylori* is involved in the pathogenesis of any EBV-positive tumors remains to be determined. Note, however, that there are small numbers of gastric carcinomas arising in individuals with a history either of gastritis cystica profunda, a rare benign lesion thought to reflect previous injury to the mucosa (159), or of partial gastrectomy (so-called remnant stump carcinoma) (156). In both of these situations, where chronic inflammation likely occurs for reasons other than *H.pylori* infection, the EBV association rate is elevated to above 30%. Gastric carcinoma is a major malignancy that affects all populations across the globe without the kind of marked geographic variation seen for NPC. Therefore, taking a mean virus association rate of 10% suggests that slightly more than 80,000 EBV-positive gastric carcinomas arise worldwide each year (7), a cancer burden which is at least the equal of that attributed to the more celebrated nasopharyngeal tumor.

EBV's role in the pathogenesis of gastric carcinoma is as yet poorly understood. Again it seems likely that local inflammation increases the chance of the virus being acquired via lytic reactivation from the B cell reservoir, and perhaps pre-neoplastic changes render gastric epithelial cells susceptible either to infection *per se* or to retention of the virus genome. At presentation, viral gene expression in tumor cells is consistently either Latency I or, in the up to 50% cases where LMP2 transcription is detectable, an intermediate Latency I/II (160). Crucially LMP1 is not detectable; however several reports have noted aberrant expression of a lytic cycle-associated protein, BARF1 (161), which in secreted form can act as a decoy colony stimulating factor CSF1 receptor and has pro-proliferative functions in *in vitro* models of epithelial growth (162). Mechanisms of malignant change appear to be different from those seen in NPC. The tumor has fewer gross chromosomal aberrations and lower overall rates of somatic mutation, although particular genes such as ARID1A (encoding a subunit of the SWI/SNF chromatin remodeling complex) and PI3KCA (encoding a key regulator of the PI3K/Akt signaling pathway) are among the few showing relatively frequent mutation (163). Most importantly, EBV-positive gastric cancer is distinguished from other forms of the tumor by its hyper-methylated genome; indeed, to many observers, it is this distinct epi-genotype that firmly establishes the EBV-positive tumor as a pathogenetically distinct subgroup of gastric carcinomas (164, 165). Developing an idea first raised in the context of NPC (166), it has been proposed that EBV actively drives oncogenic change through epigenetic modification of the host cell genome, in particular through targeting tumor suppressor genes such as p16 and E-cadherin known to be epigenetically silenced in virus-positive gastric carcinoma (167).

### Leiomyosarcomas of the Immunocompromised

The profound immune impairment associated with late-stage AIDS, particularly as seen in HIV-positive infants, has produced another unexpected example of EBV's oncogenic potential if, by chance, the virus infects an atypical target cell. Leiomyosarcoma, a smooth muscle cell tumor, is extremely rare in immunocompetent individuals where it shows no association with EBV. However, incidence of the tumor is raised in late stage AIDS, particularly in children in the pre-HAART era, and all these AIDS-associated cases proved to be EBV genome-positive (168), as are occasional tumors of the same type arising in iatrogenically immunosuppressed (169, 170) or congenitally immunocompromised patients (171–173). Limited analysis by staining for viral antigens suggests an atypical EBNA2+, LMP1-, LMP2A+/– infection (169–173) which, like the virus' contribution to tumor growth, remains to be fully investigated.

## The Search for Common Themes in EBV-associated Oncogenesis

The above overview highlights the unique status of EBV as a tumor virus linked to an unprecedentedly wide range of tumors of B cell, T/NK cell, epithelial and smooth muscle cell origin. It is tempting to search for common features underlying all these diseases, but this may be a fool's errand. Even comparisons between the different tumors of B cell origin show that EBV's pathogenetic role is highly context-dependent. Tumors of different types express different combinations of EBV latent gene products and are characterized by different patterns of genetic and epigenetic change. As outlined below, however, there are some interesting links between individual tumor types that should be further explored.

### Inflammation and the Tumor Microenvironment

One notable example is the likely involvement of inflammatory precursor lesions in the development of the T/NK lymphomas, NPC and gastric carcinoma (19). In the case of T/NK cell disease, inflammation is very probably driven by cytokines/chemokines produced by the infected T/NK cells themselves; in contrast, the epithelial tumors may require an independent inflammatory stimulus, such as inhaled particles/allergens in the case of NPC and either *H. pylori* infection or physical damage to the mucosa in the case of gastric carcinoma. *In vitro* models, for example using organoid cultures, may be a useful first step in studying how inflammatory signals influence the outcome of EBV infections in normal nasopharyngeal or gastric epithelium.

A related issue, concerning the tumor itself rather than precursor lesions, is the likely importance of the microenvironment within which the malignant cells lie (174). This is particularly significant for those tumors, namely HL, NPC and gastric carcinoma, that have marked non-malignant cell infiltrates. Multiple immune cell types make up the infiltrate, at least some of which might reflect an on-going virus-specific or tumor-specific response. In general, T cells are the most common infiltrating cell type (174), far outnumbering NK cells or B cells. However, in the absence of data from EBV antigen-specific markers such as MHC-tetramers, the significance of their presence within the tumor remains unclear (23). In the case of HL and especially gastric carcinoma, CD8+T cells tend to be more numerous in virus-positive compared to virus-negative tumor sections, implying the infiltration of virus-specific effectors; however, these T cells are not necessarily in contact with tumor cells. Indeed in the case of HL, the Reed-Sternberg cells are often surrounded by a rosette of CD4+ T cells, apparently attracted through cytokine ligand/receptor interactions and more likely reflecting pro-survival/immunosuppressive signaling than immunologic attack (175).

### Immune Surveillance, Tumor Development, and Immunotherapy

While the tumor microenvironment may be immunosuppressive by the time the malignant clone appears, for at least two of the above tumors, HL and NPC, there is strong circumstantial evidence that precursor lesions have been subject to immune control at some point during tumor evolution. Firstly, a mix of epidemiological data and genome-wide association studies have shown that HLA class I type does influence tumor risk. In the West, one of the commonest Caucasian HLA I alleles, A^*^0201, is protective against EBV-positive HL while another, A^*^0101, is linked to increased risk, the effect being most marked in individuals homozygous for these HLA-A alleles (176). In S.E. Asian people, the highly prevalent A^*^1101 allele is protective against NPC, while a common haplotype, HLA-A^*^0207, Bw^*^4601 increases risk; indeed HLA I gene polymorphism has by far the strongest association with NPC susceptibility in genome-wide studies (131, 132). These findings, bolstered by the observation that A^*^0201 and A^*^1101 mediate the strongest responses to CD8+ T cell epitopes in LMP2, suggest that virus-specific CD8+ T cell surveillance does play a protective role at some stage in the pathogenesis of these tumors (23). This is further supported by the fact that in up to 30% cases both EBV-positive HL (177) and NPC (178) appear to have either partially or completely lost HLA I expression, a finding which for NPC has recently been validated by genomic studies showing mutation of either individual HLA I alleles or pan-HLA pathway genes (142). Parallel studies that focus on the EBV-positive subset of gastric cancers are awaited with interest.

Most EBV-positive tumors nevertheless retain some or all HLA I expression at the point of presentation. This raises the question as to what extent such tumor cells are still susceptible to virus-specific T cell recognition. BL cells are at one end of the spectrum in this respect; they are surface HLA I-positive yet are protected from virus-specific CD8+ T cell recognition by an antigen processing defect that appears inherent to the tumor (51, 179). At the other end are the B-LPD lesions; they express the full range of EBV latent proteins and are clearly sensitive to such recognition, as shown by the ability of EBV-specific (predominantly CD8+) T cell preparations to cure B-LPD disease in immunocompromised patients (180). Analogous immunotherapeutic approaches, often using T cells specifically targeting the Latency II antigens EBNA1, LMP1, and LMP2, have had more limited success against HL (181) and NPC (182), emphasizing the more complex evolution of these tumors and their acquisition of multiple immune evasion strategies. Besides HLA I downregulation, other evasive influences shared by HL (183, 184), NPC (185), gastric carcinoma (186), and most recently T/NK cell disease (187) include infiltration by myeloid-derived suppressor cells (MDSCs), secretion of immunosuppressive cytokines such as IL10, and high level expression of the T cell-inhibitory programme death ligand (PDL)-1 (174). Each of these represent potential targets for therapy with the PDL1-PD1 checkpoint attracting most current attention. Interestingly, treatment of HL with anti-PDL1 blocking antibodies is indeed proving valuable in the treatment of HL (188), whereas early trials in the context of NPC are giving mixed results with positive responses not always correlating with the PDL1 or HLA I status of tumor cells (189, 190).

### Understanding EBV's Oncogenic Role, New Developments, and Opportunities

New areas of interest common to different EBV-positive tumor types are also emerging. Firstly the recognition of a hyper-methylated EBV genome as a defining characteristic of virus-positive gastric carcinoma (164, 165) is awakening/reawakening interest in the methylation status of other tumors, especially NPC and the T/NK lymphomas (116) and in EBV's potential as a driver of epigenetic change; in this context, both LMP1 (191) and LMP2A (166) have been implicated as activators of DNA methyl transferases in epithelial cell systems. Another very interesting finding is the frequent presence of EBV deletion mutants in T/NK disease settings (108). This is reminiscent of earlier reports where specific deletions of the EBNA2 gene in BL (82) and of the EBNA3B gene in DLBCL (192), though only seen in a minority of tumors, appear to have had pathogenetic significance. The degree to which tumors carry mutated EBV genomes, and whether such mutations may be shared between tumor types, is an important area for future work.

While EBV's status as an oncogenic virus is now widely accepted, it should be stressed that several of the EBV-associated tumors can also present in a virus-negative form; in such cases therefore, there are alternative routes to malignancy, only one of which involves the virus. This has its advantages since it make possible comparative genomic studies that have great potential to identify differences between EBV-positive and EBV-negative tumors and thereby reveal cellular genetic changes that the virus renders redundant. Such work is currently in progress with BL (79) and its extension to HL should be equally valuable. On the other hand, having virus-positive and negative forms of essentially the same tumor does complicate epidemiological studies and, without widespread screening for virus status, makes it difficult to determine the true incidence of virus-associated disease in different geographic/racial settings. This is particularly true at the moment for tumors such as BL and gastric carcinoma, where accurate world maps of EBV-positive vs. EBV-negative disease incidence could hold important lessons for our understanding of disease pathogenesis.

By contrast, there are two tumor types that are 100% EBV-positive worldwide, undifferentiated NPC and the T/NK lymphomas; in these cases, we know that virus infection is necessary (though itself not sufficient) for tumor development. One can therefore use the incidence rates of these tumors with confidence firstly to determine the global burden of these EBV-positive diseases and secondly to look at geographic/racial differences in their incidence. In that regard, both NPC and the T/NK lymphomas have fascinating epidemiologies. Both occur at highest frequency among Asian people, but there are important differences of detail. NPC is prevalent throughout South-East Asia, particularly in people of Southern Chinese descent, while the T/NK diseases are more common throughout East Asia, particularly in countries such as Japan and Vietnam, as well as in the native people (themselves of East Asian origin) of South and Central America. As described earlier, multiple sources of evidence (migration studies, familial clustering and genomics) have shown that host genetics is one factor contributing to NPC risk in Southern Chinese people, though to what extent lifestyle/environmental factors indigenous to that culture can explain the rest of their heightened risk remains open to debate. By contrast there is as yet no obvious familial susceptibility or lifestyle factor underlying the high incidence of T/NK cell diseases in East Asia.

### The Existence of “High Risk” EBV Strains?

Given these uncertainties, virologists have long entertained the idea that geographic differences in circulating EBV strains may be relevant to tumor incidence rates. Not surprisingly, because EBV has been co-evolving with our species over hundreds of thousands of years, different racial groups (that have been largely isolated from one another since migration out of Africa) now carry viruses with slightly diverged sequences. As a result there are allelic polymorphisms, particularly in EBV latent gene sequences, that can distinguish between strains of EBV prevalent in Asian vs. African vs. Western (Caucasian) populations (193). Whether Asian strains, for example, are as a group “more oncogenic” than Western strains, or whether particular strains within the Asian group are “more oncogenic” than others, are familiar questions that are now of increasing interest in the age of rapid whole genome sequencing (194, 195). For example, high throughput EBV genome sequencing, using a hybrid capture protocol to enrich viral sequences within healthy donor blood/throat washings, should quickly reveal the range of virus strains circulating in any host population; this can then be compared with the range of strains seen in EBV-positive tumors from patients within that population to look for evidence of selectivity. Interestingly, recent studies of this kind focusing on NPC have identified two polymorphisms in BALF2, an early lytic gene encoding a component of the viral DNA replication machinery, that are relatively common among virus strains prevalent in Southern China and, within that population, are associated with a 6-fold increase in NPC risk (196). Understanding how such markers of oncogenic potential might affect viral function becomes the next step. In that context, the ability to clone and manipulate virus genomes as bacterial artificial chromosomes and then to rescue these as infectious virus paves the way for laboratory studies of virus tropism and of virus gene expression in infected cell types. Current studies in this direction are already showing that individual virus strains vary in their relative tropism for B cells vs. epithelial cells and in the relative balance of latent vs. lytic infection in different settings. The evidence so far, albeit based on small numbers, suggests that viruses cloned and rescued from NPC and gastric carcinomas are indeed better able to infect epithelial cells than are viruses of IM or B lymphoma origin (197). However, much more work remains to be done to assess the broader significance of these findings and to identify the genetic basis of the differences observed.

### EBV-Associated Tumors and the Case for an EBV Vaccine

Finally, we return to the global impact of EBV-associated tumors (Table 1) in order to emphasize the size of the challenge and the need for a collective response. If one looks at the other human cancer viruses, there are now effective vaccines against the high-risk HPV types and also against HBV; at the same time, chronic HCV infection can be controlled by anti-viral drugs. If and when applied on a global scale, these measures have the potential to reduce the global burden of virus-associated cancers by more than 50%. Clearly now EBV, with its 200,000 new cancer cases per year, is the next target. After decades of relative neglect, progress toward a prophylactic EBV vaccine is now advancing at pace (7) among the academic research community and is beginning to interest pharmaceutical companies. The aim of preventing EBV infection can first be tested in seronegative young adults at risk of IM before rolling out to such high risk groups as young children in NPC families. Though there are many logistic hurdles to overcome beyond that, the long-term goal of a vaccine that could eliminate all EBV-associated diseases worldwide is too important to ignore.

## Author Contributions

All authors listed have made a substantial, direct and intellectual contribution to the work, and approved it for publication.

### Conflict of Interest Statement

The authors declare that the research was conducted in the absence of any commercial or financial relationships that could be construed as a potential conflict of interest.
